# Overexpression of microRNA-29b inhibits epithelial-mesenchymal transition and angiogenesis of colorectal cancer through the ETV4/ERK/EGFR axis

**DOI:** 10.1186/s12935-020-01700-2

**Published:** 2021-01-06

**Authors:** Yin Leng, Zhixian Chen, Hui Ding, Xiaoxu Zhao, Li Qin, Yunlong Pan

**Affiliations:** 1 Department of Gastrointestinal Surgery, The First Affiliated Hospital of Jinan University, No. 601, Huangpu Avenue, Guangzhou, 510632 Guangdong People’s Republic of China; 2 Department of Oncology, Fuda Cancer Hospital, Jinan University, Guangzhou, 510665 People’s Republic of China; 3 Medical Department, The First Affiliated Hospital of Jinan University, Guangzhou, 510632 People’s Republic of China; 4 Department of Histology and Embryology, Medical School of Jinan University, Guangzhou, 510632 People’s Republic of China

**Keywords:** Colorectal cancer, MicroRNA-29b, ETV4, ERK signaling pathway

## Abstract

**Background:**

Recent studies have reported the involvement of microRNA-29 (miR-29) family members in human cancers through their ability to regulate cellular functions. The present study investigated biological function of miR-29b in colorectal cancer (CRC).

**Methods:**

CRC tissues and adjacent normal tissues were collected and the expression of ETV4 and miR-29b in the tissues were identified. The relationship between ETV4 and miR-29b or ETV4 expression and the EGFR promoter was identified using dual-luciferase reporter gene and CHIP assays. The proliferation, invasion, migration, and apoptosis of CRC HCT116 cells were assayed using MTT assay, Scratch test, Transwell assay, and flow cytometry, respectively. Also, expression of epithelial-mesenchymal transition (EMT) markers, angiogenic factors, and vasculogenic mimicry formation were evaluated using RT-qPCR and Western blot.

**Results:**

ETV4 was upregulated, while miR-29b expression was decreased in CRC tissues. ETV4 was identified as a target gene of miR-29b, which in turn inactivated the ERK signaling pathway by targeting ETV4 and inhibiting EGFR transcription. Transfection with miR-29b mimic, siRNA-ETV4, or ERK signaling pathway inhibitor U0126 increased expression of E-cadherin and TSP-1, and CRC cell apoptosis, yet reduced expression of ERK1/2, MMP-2, MMP-9, Vimentin, and VEGF, as well as inhibiting EMT, angiogenesis, and CRC cell migration and invasion. The EMT, angiogenesis and cancer progression induced by miR-29b inhibitor were reversed by siRNA-mediated ETV4 silencing.

**Conclusions:**

miR-29b suppresses angiogenesis and EMT in CRC via the ETV4/ERK/EGFR axis.

## Background

Colorectal cancer (CRC) is the most frequently occurring malignant tumor in the gastrointestinal tract and the third leading cause of cancer-related deaths globally [1]. Over 1.2 million people worldwide suffer from this condition, which claims approximately 600,000 lives annually. Statistics have highlighted that males are more susceptible to CRC than females [2]. Although CRC can occur at any age, the occurrence of the disease rises greatly with increasing age, and most commonly occurs in individuals over the age of 50 years [3]. Other risk factors related to lifestyle and dietary factors include high consumption of red or processed meat, obesity, low levels of physical activity, and smoking, all of which are significant risk factors for the occurrence and development of CRC [4]. Moreover, CRC generally has an unfavorable prognosis, with 5-year relative survival of less than 65% in high-income countries, such as Australia, Canada, the USA, and several European countries, while remaining less than 50% in low-income countries [2]. Taken together, these deficiencies call for novel biomarkers enabling better diagnoses and prognoses for patients with CRC.

MicroRNAs (miRNAs or miRs) are small non-coding RNA molecules that have been widely demonstrated to play an important role, not only in human cancers but also in other health disorders [5]. Compelling evidence has extensively reported the functional role of several miRNAs such as miR-29b and miR-21 in the pathogenesis of CRC [6, 7]. Particularly, miR-29b, which is a member of the miR-29 family, is critically involved as a tumor suppressor in CRC [7], and has been highlighted as a novel prognostic marker for the disease [8]. A previous study has revealed that the transcription factor E26 transformation-specific-1 (ETS1) could affect disease progression in patients with non-small-cell lung cancer by up-regulating expression of miR-29b in the immune-evasion disease subtype [9]. Moreover, miR-29b and ETS1 play fundamental roles in human adipocyte metabolism and differentiation induced by adipose tissue-derived stromal cells [10]. ETS variant 4 (ETV4), a well-known member of the PEA3 subfamily of ETS-domain transcription factors, has also been reported to be involved in tumor metastasis in multiple types of cancers [11]. On the other hand, phosphorylation of ETV4 at Ser73 could suppress the ETV4 ubiquitination degradation through extracellular signal-regulated kinase (ERK) in CRC [12]. However, the inhibition of ERK provoked a reduction in the mRNA expression of ETV4 in melanoma cells occurs [13]. Despite these conflicting findings, the activation of the ERK signaling pathway can result in epithelial-mesenchymal transition (EMT) in CRC [14].

Therefore, we hypothesized that the miR-29b/ETV4/ERK network could be involved in development of CRC.

## Materials and methods

### Ethics statement

This study was conducted under the approval of the Ethics Committee of the First Affiliated Hospital of Jinan University in strict accordance with the *Declaration of Helsinki*. All patients and/or legal guardians were informed prior to experiments and signed written informed consents.

### Microarray-based gene expression profiling

The Gene Expression Omnibus (GEO) database (https://www.ncbi.nlm.nih.gov/geo/) was used to retrieve the datasets of differentially expressed genes (DEGs) related to CRC, and the datasets GSE89076 and GSE41328 were selected for DEG screening. Search results consisted of 13 CRC tissue samples and 12 normal colon tissue samples in the GSE89076 dataset, and 10 CRC tissue samples and 10 normal colon tissue samples in the GSE41328 dataset. The gene annotation platforms for GSE89076 and GSE41328 were GPL16699-Agilent-039494 SurePrint G3 Human GE v2 8 × 60K Microarray 039381 (Feature Number version) and GPL570-[HG-U133_Plus_2] Affymetrix Human Genome U133 Plus 2.0 Array, respectively. The Affy package of R language [15] was used to conduct standardized pretreatment for genes while the Limma package [16] was used to screen the DEGs in CRC tissues and adjacent normal tissues with screening criteria of |log2FC| > 2.0 and *adj.P.Val* < 0.05, after which a heatmap of DEGs was drawn. The Jvenn database (http://jvenn.toulouse.inra.fr/app/example.html) was used to analyze the intersected DEGs of the two datasets due to its ability to compare and analyze the datasets of different elements using Venn diagram [17]. The two gene-disease relational databases Digsee (http://210.107.182.61/geneSearch/) and DisGeNET (http://www.disgenet.org/web/DisGeNET/menu/search?4) were used for searching the CRC-related genes with “Colorectal Carcinoma” as the keyword. Results were compared with those in Jvenn, whereas the protein association information was provided by the String database (https: //string-db.org/). Cytoscape 3.6.0 software [18] was used to identify the gene interaction information of the DEGs related to CRC, the disease genes, and to establish a protein-protein interaction (PPI) network. The TargetScan (http://www.targetscan.org/vert_71/), DIP (http://ophid.utoronto.ca/mirDIP/), miRWalk (http://mirwalk.umm.uni-heidelberg.de/), microRNA (http://34.236.212.39/microrna/getGeneForm.do), and miRpath (http://lgmb.fmrp.usp.br/mirnapath/tools.php) were the five tools used for predicting the miRNA-mRNA relationship, which was used to predict the potential miRNAs that regulated ETV4, whereupon the predicted results were compared by Jvenn. The target genes of miR-29b were predicted by a bioinformatics website (http://www.microRNA.org).

### Study subjects

A total of 76 cancer tissue samples and 76 adjacent normal tissue samples (no cancer tissues following pathological examination) were collected from patients (aged between 37 and 71 years, with a mean age of 59.11 ± 6.60 years), who were initially diagnosed with CRC at the First Affiliated Hospital of Jinan University between September 2018 and September 2019. CRC tissue samples served as the CRC group and for pathological examination, and the adjacent normal tissue samples were used as the normal control group. Based on the American Joint Committee on Cancer (AJCC) tumor node metastasis (TNM) staging system (2019) [19], the CRC tissues from the 76 patients were classified as follows: 10 cases at stage I, 23 cases at stage II, 26 cases at stage III, and 17 cases at stage IV. The inclusion criteria: patients were confirmed with CRC according to clinical and imaging manifestations and pathological examinations [20]; patients had received the standard diagnosis and treatment procedures; patients had no history of treatment with radiotherapy or chemotherapy before operation. The exclusion criteria: patients with a non-standard diagnosis or treatment procedures; patients having a history of other malignancies. The tumor and adjacent normal tissue samples were cut into small pieces and quickly cryopreserved at − 80 °C.

### Hematoxylin-eosin (HE) staining

All specimens were fixed in formaldehyde, dehydrated, embedded with paraffin, cut into 4 µm serial sections, and stained with HE. In brief, the sections were dewaxed twice with xylene (15 min/time), rehydrated twice with absolute ethyl alcohol (5 min/time), 90% ethanol for 5 min, and 80% ethanol for 5 min. Then, the tissue sections were stained with hematoxylin for 5 min, washed with water, rapidly differentiated with 1% hydrochloric acid ethanol, and washed with water again. The sections were then dehydrated with 80% ethanol for 5 min, 90% ethanol for 5 min, twice with absolute ethyl alcohol (5 min each time), cleared twice with xylene (15 min/time), and mounted with neutral gum. The sections were then observed and photographed under a light microscope. Visual fields were randomly selected and analyzed using the morphological image analysis system (JD-801, Nanjing Jetta Technology, Nanjing, China). The experiment was repeated three times independently. According to the tumor regression grade (TRG) scoring systems, the scoring of the degree of pathological changes was as follows: 0 points indicated complete regression, with no tumor cells visible under the microscope; 1 point indicated a nearly complete regression, with only single or small clusters tumor cells; 2 points indicated partial regression, with significant regression, but more abundant residual tumors than single or small clusters of tumor cells; 3 points indicated poor or no regression, with a wide range of residual tumors and no obvious regression.

### Immunohistochemistry

Immunohistochemistry was employed to detect expression of ETV4 in epithelial cells in the cancer and adjacent normal tissue samples. Tissue samples were fixed with 10% formaldehyde, embedded in paraffin, and cut into 4 µm serial sections. The sections were subsequently heated at 60 °C for 1 h, dewaxed in xylene, dehydrated with gradient ethanol, boiled in 0.1 M sodium citrate for 20 min, cooled on the lab bench, and washed 3 times with 0.2 M phosphate-buffered saline (PBS) (5 min/time). Following inactivation with 3% catalase and washing with 0.2 M PBS for 3 times (five min each time), the sections were rinsed 3 times with 0.2 M PBS (pH 7.4, 5 min/time), dried, and further blocked with 5% bovine serum albumin (BSA) at 37 °C for 30 min. Next, the sections were incubated with mouse monoclonal antibody to ETV4 (1: 500, ab70425, Abcam, Cambridge, UK) [21] at 4 °C overnight, followed by 3 washes with 0.2 M PBS (pH 7.4, 5 min/time) on the following day. After the sections were dried, they were incubated with the biotinylated secondary goat anti-mouse immunoglobulin G (IgG) (1: 1000, ab6789, Abcam) for 30 min at 37 °C. A color developing reagent was prepared by fully mixing one drop of A, B, and C reagents of a diaminobenzidine (DAB) staining kit (DA1010, Beijing Solarbio Science & Technology Co., Ltd., Beijing, China) and l mL distilled water respectively, mixed well, and then stored in a 4 °C freezer. Afterwards, the sections were washed 3 times with 0.2 M PBS (pH 7.4, 5 min/time), visualized by incubation with the DAB solution for 8 min in the dark, and rewashed with running water. The sections were then subjected to hematoxylin staining, dehydration, clearing, sealing, and observation with a light microscope. The cells with nucleus stained blue or light blue were identified as negative cells and those with a nucleus presenting with brownish-yellow or strongly brownish-yellow particles were regarded as positive cells. The number of positive cells was then counted using the Nikon image analysis software (Nikon Instruments Inc., Tokyo, Japan). Five high-magnification visual fields were randomly selected from each section and 200 cells were counted in each field. The percentage of ETV4-positive cells relative to the total cells in each individual field was calculated using the double-blind method and the average values were obtained. The experiment was conducted three times independently.

### Weidner counting

Conventional streptavidin-peroxidase (SP) staining was conducted with CD34 to label the vascular endothelial cells. CD34 was localized in the cytoplasm of vascular endothelial cells presenting a brownish-yellow color. Based on the Weidner counting method, three regions with the highest vessel density were identified at low magnification, and two pathologists independently counted the number of micro-vessels under high magnification. Single isolated or multiple closely spaced endothelial cell clusters with or without the presence of lumen and with clear boundaries with adjacent tumor cells and surrounding connective tissue components were counted as 1 microvessel, with the exception of the vessels with a lumen diameter exceeding that of eight red blood cells, or with smooth muscle in the tube wall. The mean number of micro-vessels in three fields was taken as the micro-vessel density (MVD). In the event of counting difference between two pathologists exceeding 10%, the number of micro-vessels was re-counted by consensus. The experiment was conducted three times independently.

### Cell grouping and transfection

CRC cell line HCT116 (iCell Bioscience Inc., Shanghai, China) was cultured in DMEM (190,040, Gibco, Grand Island, NY, USA) and seeded into a 6-well plate at 1 × 10^5^ cells/well. The cells were then incubated with 5% CO_2_ with saturated humidity at 37 °C. When cell density reached about 80–90%, they were passaged.

Cells were grouped into the following groups: blank (HCT116, without any transfection), negative control (NC) (HCT116 cells transfected with NC sequence), miR-29b mimic (HCT116 cells transfected with miR-29b mimic sequence), miR-29b inhibitor (HCT116 cells transfected with miR-29b inhibitor sequence), small interfering RNA (siRNA)-ETV4 (HCT116 cells transfected with siRNA-ETV4 sequence), miR-29b inhibitor + siRNA-ETV4 (HCT116 cells co-transfected with miR-29b inhibitor sequence and siRNA-ETV4 sequence), DMSO (solvent), and U0126 (ERK signaling pathway inhibitor) groups [22]. 24 h before transfection, the cells had been seeded into a 6-well plate. After achieving about 30–50% cell confluence, cell transfection was conducted using Lipofectamine® 2000). The serum-free Opti-minimum essential medium (Opti-MEM) (250 µL, 51,985,042, Gibco, Gaithersburg, MD, USA) was used to dilute the plasmids (100 pmol each) in the NC, miR-29b mimic, miR-29b inhibitor, miR-29b inhibitor + siRNA-ETV4, and siRNA-ETV4 groups (50 nM final concentration). Diluted plasmids were incubated for 5 min. An additional 250 µL serum-free Opti-MEM medium was used to dilute 5 µL portions of Lipofectamine® 2000, followed by incubation for 5 min. The two diluted mixtures were then mixed and incubated with cells for 20 min. After transfection, the cells were further cultured in a 5% CO_2_ incubator at 37 °C, with change to complete medium after 6 to 8 h. Next, after 24 to 28 h of further incubation, the subsequent experiments were conducted.

### Dual-luciferase reporter gene assay

Dual-luciferase reporter gene assay was carried out to verify whether ETV4 was a target gene of miR-29b. The synthetic ETV4-3’untranslated region (UTR) gene fragment was introduced into pMIR-reporter (Promega, Madison, WI, USA) through the endonuclease sites SalI and bglII. The mutation sites of complementary sequences were designed on ETV4 wild-type (WT) sequence. After cleavage with a restriction endonuclease, the target fragment was inserted into the pMIR-reporter plasmid using T4 DNA ligase. Followed by identification of positive clones, the recombinant plasmids were identified by DNA sequencing, sub-cloned into psiCHECK-2 vector, and transformed into *Escherichia coli* DH5α cells to amplify the plasmids. The plasmids were then extracted according to the instructions of the Omega kit (Promega, Madison, WI, USA), and the cells were seeded into a 6-well plate at a density of 1 × 10^4^ cells/well. NC and miR-29b mimic were co-transfected with the luciferase-reporter vectors into the HCT116 CRC cell line. The cells were then transfected and cultured for 48 h, and collected afterwards. The effect of miR-29b treatment on the luciferase activity of ETV4 3′-UTR in cells was examined according to the instructions of the dual-luciferase activity detection kit (Genecopoeia Inc., Rockville, MD), using a Glomax 20/20 luminometer (Promega, Madison, WI, USA) for detection.

To verify whether ETV4 bound to EGFR promoter region, the synthetic EGFR promoter region fragment was introduced into the pMIR-reporter (Promega, Madison, WI, USA) using the endonuclease sites SalI and bglII. The mutation sites of complementary sequences were designed based on EGFR wild-type (WT) sequence. After cleavage with restriction endonucleases, the target fragment was inserted into the pMIR-reporter plasmid using T4 DNA ligase. Then the cells were seeded into a 6-well plate at 1 × 10^4^ cells/well. Luciferase reporter vectors were cotransfected with HEK293 cells and CRC cell line HCT116 and cultured for 48 h and collected afterwards. The changes of luciferase activity in EGFR promoter region in cells were detected using dual-luciferase activity detection kit (Genecopoeia Inc., Rockville, MD) as above.

### CHIP

The HCT116 cells were fixed in 4% formaldehyde (final concentration 1%), ultrasonicated, added with Rabbit anti human ETV4 antibody to bind to EGFR promoter, and added with protein A Agarose/Salmon Sperm DNA to bind to ETV4 antibody/ETV4/EGFR promoter complex. Next, the complex was precipitated and cleaned to remove nonspecific binding. The enriched ETV4/EGFR promoter complex was obtained after elution, and then decrosslinked, purified, and analyzed by qPCR. The sequence of EGFR promoter: F (5′-ccaggtacggccgctgctgtg-3′) and R (5′-cgcgagachadacgcctctcttcttt-3′).

### RT-qPCR

Total RNA was extracted using a RNA extraction kit (10,296,010, Invitrogen). RNA was reversely transcribed into complementary DNA (cDNA) using Primescript™ RT reagent kit (RR014A, Takara Bio Inc., Beijing, China). The primers were designed and then synthesized by Takara Bio Inc. (Table 1). RT-qPCR was performed using PCR Kit (KR011A1, Tiangen Biotech Co., Ltd., Beijing, China). U6 was used as the internal reference for miR-29b whereas glyceraldehyde-3-phosphate dehydrogenase (GAPDH) was taken as an internal reference for evaluation of ETV4, ERK1/2, matrix metalloproteinase (MMP)-2, MMP-9, E-cadherin, Vimentin, vascular endothelial growth factor (VEGF), and tumor suppressor region 1 (TSP-1). Expression of each gene in each group was calculated by the 2^−ΔΔCt^ method.

**Table 1:** Primer sequences for RT-qPCR

Gene	Primer sequence
miR-29b	F: 5′-TGAACCTTTGTCTGGGCAACT-3’
	R: 5′-TGGTATCCTTGAGGGATTGGTTC-3′
ETV4	F: 5′-CACTCCCCTACCACCATGGA-3′
	R:5′-GGACTTGATGGCGATTTGTC-3′
ERK1	F: 5′-CAGGAGACAGCACGCTTCCAG-3′
	R: 5′-TCTAACAGTCTGGCGGGAGAGG-3′
ERK2	F: 5′-GCTGTTCCCAAATGCTGACT-3′
	R: 5′-CTCGTCACTCGGGTCGTAAT-3′
MMP-2	F: 5′-AGTTTCCATTCCGCTTCCAG-3′
	R: 5′-CGGTCGTAGTCCTCAGTGGT-3′
MMP-9	F: 5′-GAAGATGCTGCTGTTCAG-3′
	R: 5′-AAATAGGCTTTCTCTCGGTA-3′
E-cadherin	F: 5′-GACCAAGTGACCACCTTA-3′
	R: 5′-AGAGCAGCAGAATCAGAAT-3′
Vimentin	F: 5′-CATTGAGATTGCCACCTAC-3′
	R: 5′-TCGTTGATAACCTGTCCAT-3′
VEGF	F: 5′-CCATGAACTTTCTGCTCTCTTG-3′
	R: 5′-GGTGAGAGGTCTAGTTCCCGAA-3′
TSP-1	F: 5′-GTGTTTGACATCTTTGAACTC-3′
	R: 5′-CCAAAGACAAACCTCACATTC-3′
GAPDH	F: 5′-GGTATCGTGGAAGGACTC-3′
	R: 5′-GGGATGATGTTCTGGAGAG-3′
U6	F: 5′-GTGCTCGCTTCGGCAGCACATATAC-3′
	R: 5′-AAAAATATGGAACGCTTCACGAATTTG-3′

miR-29b, microRNA-29b; RT-qPCR, reverse transcription quantitative polymerase chain reaction; ETV4, ETS variant 4; ERK, extracellular regulated protein kinases; MMP, matrix metalloproteinase; VEGF, vascular endothelial growth factor; TSP-1, thrombospondin 1; GAPDH, glyceraldehyde-3-phosphate dehydrogenase; F, forward; R, reverse

### Western blot analysis

Total protein was extracted using radioimmunoprecipitation assay (RIPA) lysis buffer (R0010; Beijing Solarbio Science & Technology Co., Ltd., Beijing, China). The protein was separated by polyacrylamide gel electrophoresis (PAGE) and transferred onto nitrocellulose membrane using the wet method. The membrane was blocked with 5% BSA for 1 h and then probed at 4 °C overnight with mouse anti-human monoclonal primary antibodies to ETV4 (1:500, ab70425, 55 KD [21]), EGFR (1:1000, ab52894, 175 KD), MMP-2 (1:1000, ab97779, 75 KD [23]), E-cadherin (1:50, ab76055, 97 KD [24]), MMP-9 (1:1000, ab58803, 92 KD [25]), Vimentin (1:1000, ab8978, 57 KD [26]), VEGF (1:1000, ab9540, 34 KD [27]), TSP-1 (1:1000, ab1823, 150 KD [28]), and p-ERK1/2 (1: 1000, ab54230, 44/42 KD [29]), with GAPDH (1: 2000, ab8245, 36 KD) serving as the internal reference. After three PBS washes (10 min each), the membrane was incubated with horseradish peroxidase (HRP)-labeled goat anti-mouse IgG (Abcam, 1:10,000, ab6785), re-washed with the Tris-buffered saline Tween-20 (TBST) three times (10 min each), developed with enhanced chemiluminescence (ECL) solution (WBKLS0500, Pierce, Rockford, IL, USA), and photographed for analysis. All antibodies were obtained from Abcam. The gray value was analyzed using the Alpha Digi-Doc system (Alpha Innotech), and the ratio of the gray value of the target band to that of GAPDH depicted the relative protein expression.

### 3-(4,5-dimethylthiazol-2-yl)-2, 5-diphenyltetrazolium bromide (MTT) assay

After 48 h of transfection, the HCT116 cells in a logarithmic growth phase were seeded into a 96-well plate at 1 × 10^4^ cells/well, with 200 µL medium each well. After 24 h of cell adherence, the cells were incubated in a 5% CO_2_ incubator at 37 °C for 24, 48, or 72 h. Thereafter, each well was added with 20 µL MTT (5 mg/mL) and incubated for 4 h at 37 °C, followed by aspiration of the supernatant. Afterward, 150 µL dimethyl sulfoxide (DMSO) was added to each well and the plates were shaken for 10 min. The optical density (OD) value of each well at 450 nm was then evaluated using a microplate reader (Multiskan FC, Thermo Fisher Scientific Co., Ltd., New York, USA).

### Three-dimensional cell culture and vasculogenic mimicry (VM) formation assay

The mixture of culture medium, NaOH (0.1 M), and type-I collagen (Shengyou Biotechnology Co. Ltd., Hangzhou, China) was added into a 6-well plate with 200 µL per well and the plate was allowed to stand for 15 min for subsequent experiments. The cell lines were cultured in different culture medium containing 10% FBS at 37 °C with 5% CO_2_. After cell confluence reached 80%, the cell density was adjusted to 8 × 10^5^ cells/mL, and the cells were seeded into a 6-well plate with 1 mL portions per well. The VM formation was observed using an inverted microscope every day of the subsequent indication. Additionally, Periodic acid-Schiff’s (PAS) staining was performed for the observation of VM formation on the 6th day. Cells were then fixed with 10% formaldehyde for 5 min, oxidized in aqueous periodic acid solution for 5 min, and rinsed 3 times with distilled water. Thereafter, the cells were stained in Schiff’s reagent for 20 min and washed with tap water for 10 min until the cells turned red. Five fields were randomly selected under an inverted microscope and the average number of VM observed per field was regarded as the VM density [30]. The tube-like VM structures were viewed using phase-contrast microscopy and photographed. In formalin-fixed paraffin-embedded (FFPE) tissues, immunohistochemical and PAS staining was used to detect the VMs [31].

### Scratch test

At 24 h post-transfection, the cells were seeded into a 6-well plate with 1 × 10^6^ cells/well. Upon achieving approximately 95% confluence, the cells were vertically and linearly scratched with a 20 µL micropipette in the 6-well plate. Cells were then washed with D-Hanks solution to remove floating cells, cultured with serum-free culture medium, and photographed at hours 0 and 48. The cell migration distance was measured using the Image-Pro Plus Analysis software (Media Cybernetics, Silver Spring, MD, USA).

### Transwell assay

At 72 h of post-transfection, the cells were starved in serum-free medium for 12 h to remove the influence of serum constituents, and re-suspended with serum-free medium Opti-MEMI (31985-070, Invitrogen) containing 10 g/L BSA. Cell density was adjusted to 2 × 10^6^ cells/mL. First, 50 µL Matrigel (40111ES08, Sigma, St Louis, MO, USA) was evenly distributed in the chamber. After 48 h, 200 µL of single cell suspension (4 × 10^4^ cells) was added into the apical chamber in each group, followed by the addition of 650 µL Opti-MEMI (31985-070, Invitrogen) containing 10% FBS into the basolateral chamber. The chamber was then incubated with 5% CO_2_ at 37 °C for 12 h. Cells in the chamber were removed with a cotton bud and washed with PBS, followed by fixation in methanol for 30 min. The cells were then stained with 0.1% crystal violet solution for 20 min and the cells in the upper layer of the microporous membrane were carefully wiped off by cotton swabs. Finally, the invaded cells in four randomly selected visual fields were counted under an inverted microscope.

### Flow cytometry

At 72 h post-transfection, the cells were detached to adjust cell density to 1 × 10^6^ cell/mL. Next, the cell suspensions (1 mL) were centrifuged for 10 min at 403*g*, and re-centrifuged. After removing the supernatant, the cell pellet was fixed in 70% precooled ethanol overnight at 4 °C. The fixed cell suspensions (100 µL) were treated with 50 µg propidium iodide (PI) containing RNAase for 30 min in a dark room and then filtered with a 100 mesh nylon net. Flow cytometry (BD, FL, NJ, USA) was used to detect the cell cycle at 488 nm.

Annexin and V-fluorescein isothiocyanate (FITC)/PI double staining was used to detect cell apoptosis. The treated cells were then cultured for 48 h in an incubator containing 5% CO_2_ at 37 °C, centrifuged, and then suspended in 200 µL binding buffer. Afterward, the cells were added with 10 µL Annexin V-FITC (ab14085, Abcam) and 5 µL PI for 15 min of incubation in the dark followed by the addition of 300 µL binding buffer. A flow cytometer was used to detect cell apoptosis at 488 nm.

### Statistical analysis

Statistical analysis was performed using the SPSS 21.0 software. The measurement data were summarized as the mean ± standard deviation. Data between the two groups were compared by paired *t*-test, while data among multiple groups were compared using a one-way analysis of variance (ANOVA) followed by a Tukey’s multiple comparisons post-test. A comparison of data at different time points was performed using two-way ANOVA. A value of *p* < 0.05 was statistically significant.

## Results

### The functional significance of miR-29b, ETV4, and the ERK signaling pathway in CRC

**Fig. 1:**
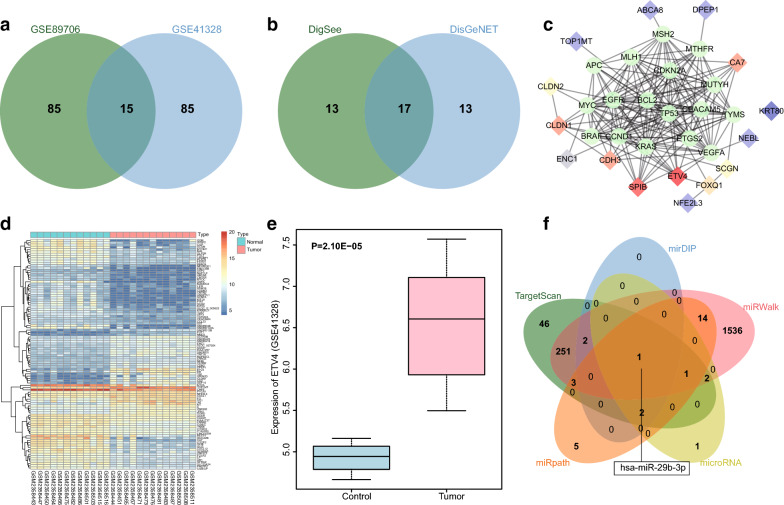
Bioinformatics analysis predicting the DEGs and their molecular interactions in CRC. **a** The intersection of the top 100 DEGs with the largest fold change between GSE89076 and GSE41328 datasets, revealing 15 intersected genes. **b** The intersection of CRC genes between Digsee and DisGeNET, showing 17 intersected genes. **c** The PPI network of DEGs and disease genes; the diamonds refer to DEGs and the circles to disease genes. **d** A heatmap of the top 100 genes with largest fold change in dataset GSE89076; the X-axis refers to sample number and the Y-axis to DEGs; the histogram in upper right indicates changes by color gradation, where each rectangle corresponds to a sample’s expression value. **e** The expression level of ETV4 in CRC and matched normal colonic tissues in dataset GSE41328. **f** The intersection of miRNAs that could bind to ETV4 predicted by the TargetScan, DIP, miRWalk, microRNA and miRpath databases, showing has-miR-29b-3p at the intersection

R language was used to screen DEGs from CRC-related gene expression datasets (GSE89076 and GSE41328) with the screening criteria of |log2FC| > 2.0 and *adj.P.Val* < 0.05. The top 100 DEGs with the largest fold change in each dataset were selected, and those from two datasets were subsequently intersected, which revealed 15 intersection genes in the Venn diagram (Fig. 1a) selected for the subsequent study. The Digsee and DisGeNET databases were applied to search the CRC-related genes, and the top 30 genes with high correlations between database were selected. Genes obtained from two databases were intersected, which yielded 17 intersection genes in the Venn diagram (Fig. 1b), which were designated as disease-related genes. According to the protein interaction information provided in the String database, the PPI network of DEG candidates and disease gene candidates was established. The PPI network (Fig. 1c) showed that the disease genes interacted closely with each other in the regulatory network and that ETV4 was the candidate presenting the highest correlation with diseased genes or other DEGs. According to the top 100 DEGs in GSE89076, the heatmap of DEGs was drawn (Fig. 1d), which showed that ETV4 was expressed higher in CRC tissues than in adjacent normal tissues. Additionally, the expression level of ETV4 in CRC and matched normal colonic tissues in GSE41328 are shown in Fig. 1e, likewise revealing that ETV4 was highly expressed in CRC tissues. Accordingly, previous studies have shown that the ERK signaling pathway was abnormally activated in CRC [32–34] and that ETV4 can activate the ERK signaling pathway [35, 36]. However, in the present study, we focused on whether the differentially expressed ETV4 affected CRC through the ERK signaling pathway. TargetScan, DIP, miRWalk, microRNA, and miRpath databases were used to predict the miRNAs that regulated ETV4. The predicted miRNAs obtained from all five databases were compared and a Venn diagram was plotted (Fig. 1f), which showed that hsa-miR-29b-3p was the only intersecting miRNA having the possibility to regulate ETV4. miR-29b is consistently indicated to negatively regulate the ERK signaling pathway [37]. According to in silico analysis, we inferred that miR-29b targets the ETV4 to regulate the ERK signaling pathway, thus affecting the development of CRC.

### Pathological changes in CRC tissues

**Fig. 2:**
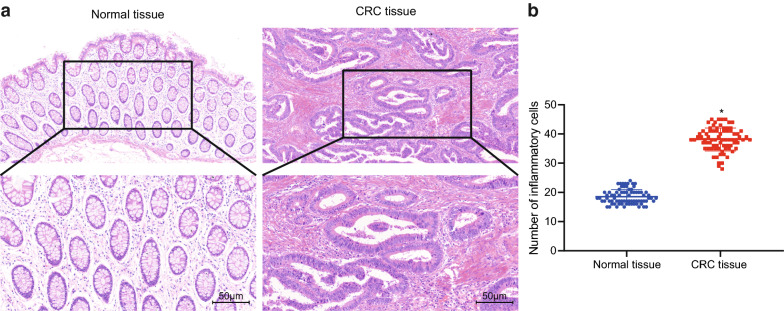
Representative images of CRC and adjacent normal tissues after HE staining. A, HE staining analysis of cancer and adjacent normal tissues (×200). B, The number of inflammatory cells. *p < 0.05 compared with adjacent normal tissues. Arrows indicate inflammatory cells. Data between the two groups were compared by paired *t*-test

Initially, HE staining was performed to compare the morphological changes between CRC tissues and adjacent normal tissues (Fig. 2a, b). We observed that secretory glands in the adjacent normal tissues had a neat and uniform arrangement. On contrary, in the CRC tissues, there was abundant inflammatory cell infiltration in the colonic mucosa and submucosa, invasion of cancer tissues into the mucosal layer, and disorganized as well as destroyed glandular tissues with ductal-like structure.

### High positive expression of ETV4 protein in CRC tissues

**Fig. 3:**
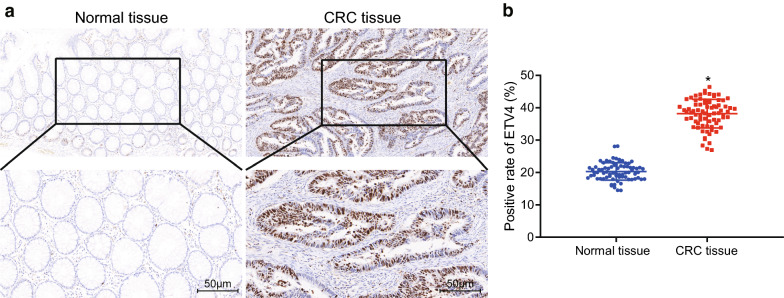
Increased positive expression of ETV4 protein in CRC tissues. **a** Immunohistochemistry analysis of adjacent normal tissues and CRC tissues (×200). **b** Positive rate of ETV4 protein in CRC tissues and adjacent normal tissues. *p < 0.05 compared with adjacent normal tissues. Data between the two groups were compared by paired *t*-test

To compare the positive expression of ETV4 protein in CRC and adjacent normal tissues, we conducted immunohistochemistry. Results showed that ETV4 positive expression presented with a brown color, while the ETV4-positive signals were mainly localized in the nucleus, with only a few in the cytoplasm (Fig. 3a). The positive rate of ETV4 protein in CRC tissues was 69.74 ± 5.23%, which was higher than that in adjacent normal tissues (32.50 ± 2.35%) (Fig. 3b). These results indicated that ETV4 protein expression was higher in CRC tissues than that in adjacent normal tissues.

### Decreased miR-29b expression and increased ETV4 expression are detected in CRC tissues

**Fig. 4:**
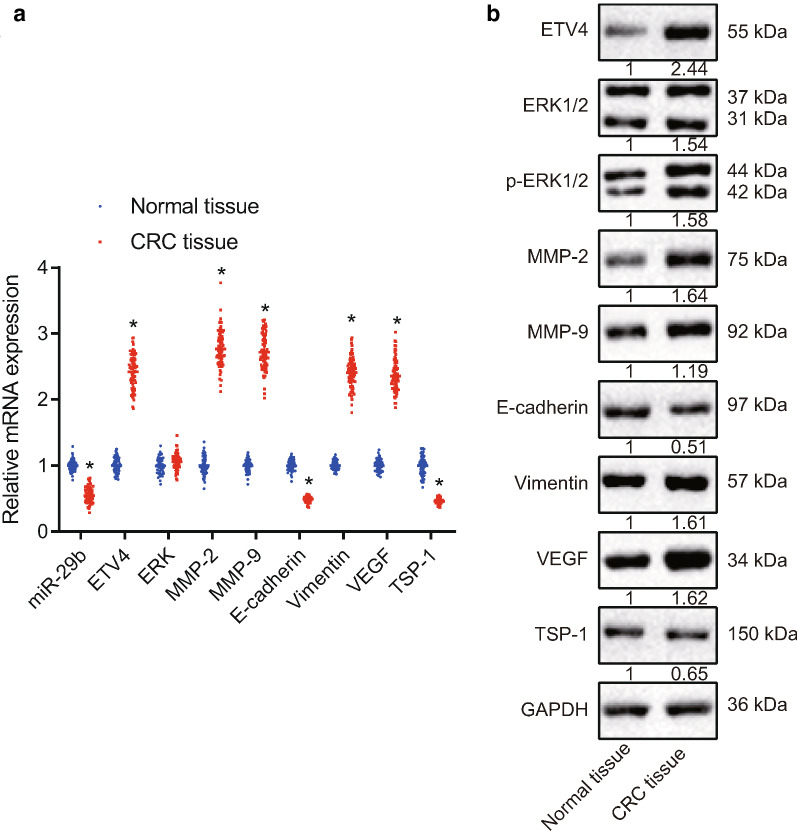
RT-qPCR and Western blot analysis results show decreased miR-29b expression and increased ETV4 expression in CRC tissues. A, miR-29b expression (normalized to U6) and mRNA expression of E-cadherin, TSP-1, ETV4, ERK1/2, MMP-2, MMP-9, Vimentin, and VEGF (normalized to GAPDH) in CRC and adjacent normal tissues determined using RT-qPCR. B, Western blot analysis of E-cadherin, TSP-1, ETV4, ERK1/2, MMP-2, MMP-9, Vimentin, and VEGF proteins (normalized to GAPDH) in CRC and adjacent normal tissues. *p < 0.05 compared with adjacent normal tissues. Each experiment was repeated 3 times independently. Data between the two groups were compared by paired *t*-test

RT-qPCR and Western blot analyses were performed to determine expression of miR-29b and ETV4 in CRC and adjacent normal tissues. RT-qPCR results (Fig. 4a) illustrated that, compared with adjacent normal tissues, CRC tissues exhibited decreased expression of miR-29b, mRNA expression of E-cadherin and TSP-1, but increased mRNA expression of ETV4, MMP-2, MMP-9, Vimentin, and VEGF, with no significant change in ERK expression. Western blot analyses results (Fig. 4b) showed that, compared with adjacent normal tissues, protein expression of E-cadherin and TSP-1 was reduced, but that of ETV4, ERK1/2, MMP-2, MMP-9, Vimentin, and VEGF was increased in CRC tissues.

### Low miR-29b expression and high ETV4 expression are associated with clinicopathological progression of CRC

After identifying expression of miR-29b and ETV4 in CRC tissues, we set out to examine their relationship with the clinicopathological features of CRC patients. Expression of miR-29b and ETV4 was measured in CRC patients with or without lymph node metastasis (LNM), with different differentiation grade and TNM stages. The results showed that miR-29b and ETV4 expressions were unrelated to age, gender, tumor size, and tumor location. Expression of ETV4 in CRC tissues with and without LNM was 2.63 ± 0.13 and 2.23 ± 0.16, respectively, indicating that its expression was somewhat increased in CRC patients with LNM. Expression of miR-29b in CRC tissues with and without LNM was 0.44 ± 0.06 and 0.65 ± 0.07, respectively, indicating that its expression was diminished in CRC patients with LNM. ETV4 expression in patients with stage I–II and III–IV CRC was 2.19 ± 0.14 and 2.60 ± 0.14, respectively, suggesting that increased ETV4 expression in CRC tissues was correlated with more advanced TNM stage. The miR-29b expression at stage I–II and III–IV were 0.67 ± 0.06 and 0.46 ± 0.07, respectively, suggesting that reduced miR-29b expression in CRC tissues was correlated with increased TNM staging (Table 2).

**Table 2:** Relationships between miR-29b expression and clinicopathological features of CRC patients

Clinicopathological feature	Case	miR-29b expression	*p*	EVT4 expression	*p*
Age (years)					
< 60	35	0.56 ± 0.15	0.476	2.40 ± 0.31	0.493
≥ 60	41	0.54 ± 0.09		2.44 ± 0.19	
Gender					
Male	54	0.55 ± 0.12	0.999	2.42 ± 0.26	0.877
Female	22	0.55 ± 0.11		2.43 ± 0.24	
Tumor location					
Colon	29	0.57 ± 0.10	0.310	2.39 ± 0.22	0.404
Rectum	47	0.54 ± 0.13		2.44 ± 0.27	
Tumor size (cm)					
< 5	41	0.53 ± 0.09	0.137	2.45 ± 0.20	0.302
≥ 5	35	0.57 ± 0.14		2.39 ± 0.30	
Differentiation grade					
Well/moderate	49	0.62 ± 0.09	< 0.001	2.28 ± 0.17	< 0.001
Poor	27	0.42 ± 0.05		2.68 ± 0.12	
LNM					
No	40	0.65 ± 0.07	< 0.001	2.23 ± 0.16	< 0.001
Yes	36	0.44 ± 0.06		2.63 ± 0.13	
TNM staging					
I/II	33	0.67 ± 0.06	< 0.001	2.19 ± 0.14	< 0.001
III/IV	43	0.46 ± 0.07		2.60 ± 0.14	

CRC, colorectal cancer; miR-29b, microRNA-29b; ETV4, ETS variant 4; LNM, lymph node metastasis; TNM, tumor node metastasis

### ETV4 is a target gene of miR-29b

**Fig. 5:**
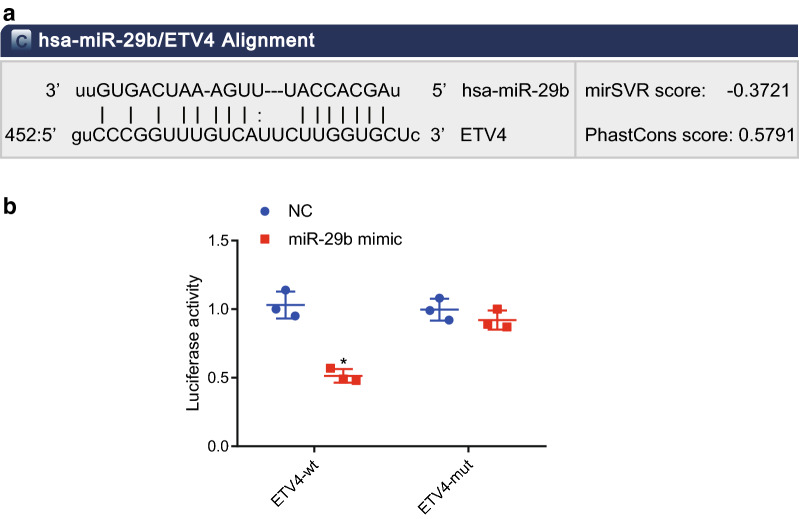
ETV4 is a target gene of miR-29b. **a** Predicted binding sites of miR-29b in ETV4 3′UTR. **b** Luciferase activity of cells transfected with ETV4-WT and ETV4-MUT in the presence of miR-29b. *p < 0.05 compared with the NC group. Each experiment was repeated 3 times independently. Data between the two groups were compared by paired *t*-test

The bioinformatics website (http://www.microrna.org) predicted binding sites between miR-29b and the 3′UTR of ETV4, suggesting that ETV4 was a potential target gene of miR-29b (Fig. 5a). Subsequently, we verified the predicted results by determining the luciferase activity. As shown in Fig. 5b, the luciferase activity of cells transfected with ETV4-WT and miR-29b mimic was lower than that in cells transfected with ETV4-WT and in NC, while no significant difference was detected in the luciferase activity of ETV4-mutant (MUT) cells. These results indicated that miR-29b was able to specifically bind to the ETV4 and down-regulate its expression.

### High MVD is detected in CRC tissues

**Fig. 6:**
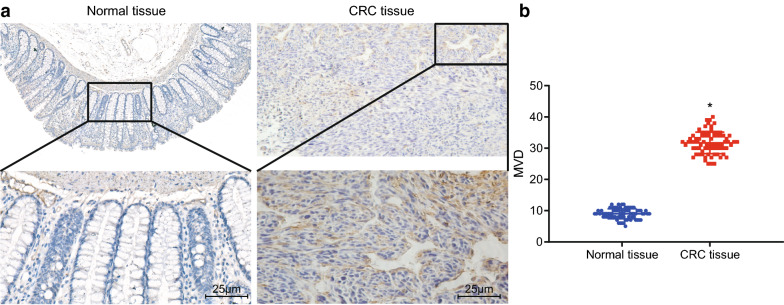
MVD is higher in the CRC tissues than in the adjacent normal tissues. **a** Representative images of CD34-positive cells in CRC tissues and adjacent normal tissues (×200). **b** MVD in CRC tissues and adjacent normal tissues calculated by Weidner counting method. *p < 0.05 compared with the normal group. Data between the two groups were compared by paired *t*-test

MVD in the CRC and adjacent normal tissues was detected using the Weidner counting method. As shown in Fig. 6a, b, CD34 was mainly expressed in vascular endothelial cells. The MVD of CRC tissues (43.62 ± 5.23) was higher than that of the adjacent normal tissues (6.88 ± 1.09). These results suggested that MVD was elevated in CRC tissues.

### Up-regulation of miR-29b reduces ETV4 expression and inhibits activation of the ERK signaling pathway

**Fig. 7:**
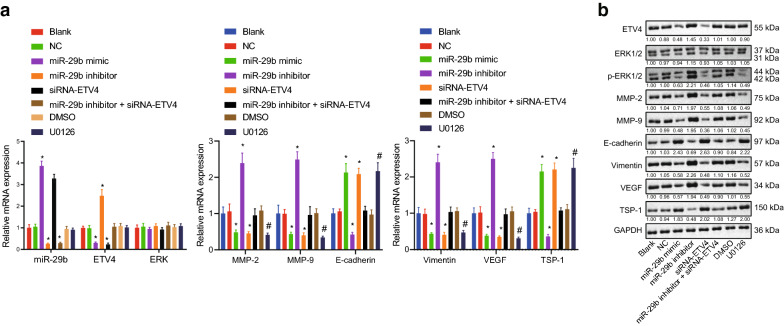
miR-29b reduces expression of ETV4 and inhibits the activation of the ERK signaling pathway. **a** miR-29b expression (normalized to U6) and mRNA expression (normalized to GAPDH) in HCT116 cells transfected with miR-29b mimic, miR-29b inhibitor and/or siRNA-ETV4 determined using RT-qPCR. **b** Western blots and quantitative analysis of ETV4, phosphorylated ERK1/2, ERK1/2, MMP-2, MMP-9, Vimentin, and VEGF proteins (normalized to GAPDH) in HCT116 cells transfected with miR-29b mimic, miR-29b inhibitor and/or siRNA-ETV4. *p < 0.05 compared with the blank and NC groups. ^#^*p* < 0.05 compared with the DMSO group. Each experiment was repeated 3 times independently. Data among multiple groups were analyzed by one-way ANOVA, followed by a Tukey multiple comparisons posttest

To study the mechanisms and the role of miR-29b and ETV4 in CRC, miR-29b mimic, miR-29b inhibitor or siRNA-ETV4 was transfected into HCT116 cells. RT-qPCR and Western blot analysis were used to measure their expression along with the ERK signaling pathway downstream gene expression in the transfected cells. Rescue experiments were also conducted by co-transfecting miR-29b inhibitor and siRNA-ETV4 into HCT116 cells. The results in Fig. 7a, b illustrated that ERK and ERK1/2 expression showed no significant difference between groups. Compared with the blank and NC groups, the miR-29b mimic group showed elevated miR-29b expression. The miR-29b mimic and siRNA-ETV4 groups showed elevated mRNA and protein expression of E-cadherin and TSP-1, and down-regulated mRNA and protein expression of ETV4, p-ERK1/2, MMP-2, MMP-9, Vimentin, and VEGF, along with reduced extent of ERK1/2 phosphorylation, but opposite changes in the aforementioned mRNAs and proteins were observed in the miR-29b inhibitor group. In the miR-29b inhibitor + siRNA-ETV4 group, there were no significant differences in expression of the aforementioned mRNAs and proteins in contrast to findings in the blank and NC groups. The changes in the aforementioned mRNAs and proteins induced by miR-29b inhibitor were reversed by siRNA-ETV4. Compared with the DMSO group, the U0126 group exhibited elevated mRNA and protein expression of E-cadherin and TSP-1, and reduced RNA and protein expression of p-ERK1/2, MMP-2, MMP-9, Vimentin, and VEGF. Collectively, overexpressed miR-29b was able to lead to a reduction in expression of ETV4 and consequently inhibited the activation of the ERK signaling pathway.

### Up-regulated miR-29b expression decreases the CRC cell growth through down-regulation of ETV4

**Fig. 8:**
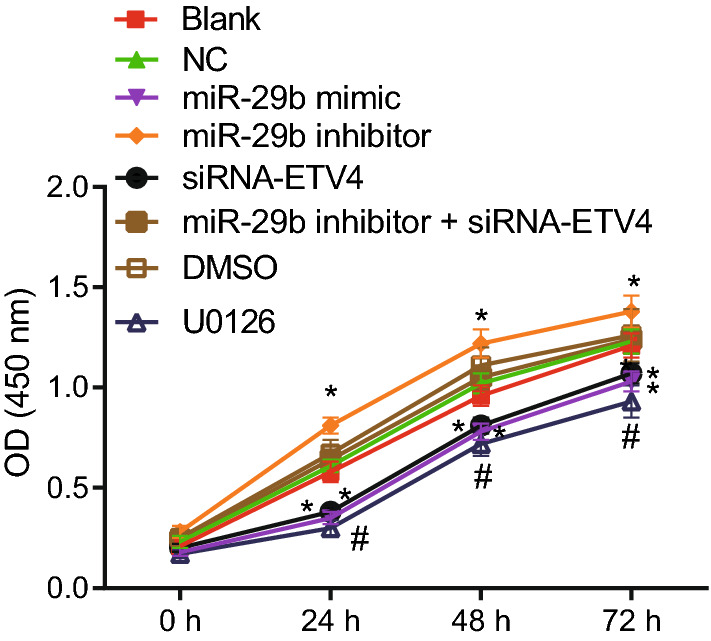
MTT results demonstrate that up-regulated miR-29b inhibits CRC cell viability by reducing expression of ETV4. *p < 0.05 compared with the blank and NC groups. ^#^*p* < 0.05 compared with the DMSO group. Each experiment was repeated 3 times independently. Data at different time points were analyzed by two-way ANOVA, followed by a Tukey multiple comparisons posttest

The MTT assay was performed to detect cell viability, where proliferating cells gave higher absorbance signals. We compared the OD values of each group to analyze the changes in cell viability. The results of MTT assay (Fig. 8) indicated that with increase of time (24, 48, and 72 h), OD values grew significantly in each group. Compared with the blank and NC groups, the cell growth in the miR-29b inhibitor + si-ETV4 group did not show any significant differences at any time point compared with the blank and NC groups; while the OD values at 24, 48, and 72 h were remarkably lower in the miR-29b mimic and siRNA-ETV4 groups, suggesting that cell growth was inhibited. However, an increase was observed in the OD values of the miR-29b inhibitor group in contrast to that in the blank and NC groups, indicating enhanced cell growth. Compared with the DMSO group, the U0126 group had reduced cell growth, with decreased OD values at 24, 48, and 72 h, suggesting that cell growth was inhibited. These results suggested that overexpressed miR-29b could retard the cell growth in CRC by targeting the ETV4.

### Up-regulated miR-29b expression inhibits VM formation of CRC cells through down-regulation of ETV4

**Fig. 9:**
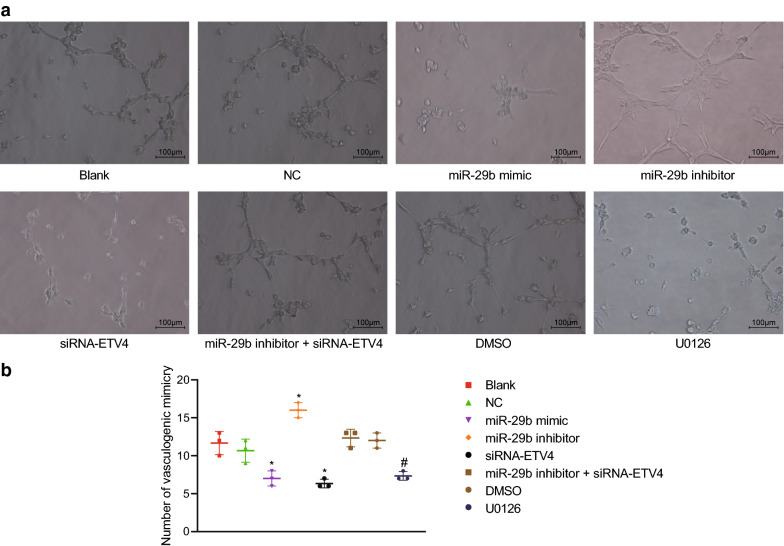
Overexpressed miR-29b inhibits VM formation of CRC cells. **a** Representative images of VM formation when CRC cells transfected with miR-29b mimic, miR-29b inhibitor and/or siRNA-ETV4 evaluated by in vitro tube formation experiment. B, The number of formed tubes. *p < 0.05 compared with the blank and NC groups (×100). ^#^*p* < 0.05 compared with the DMSO group. Each experiment was repeated 3 times independently. Data among multiple groups were analyzed by one-way ANOVA, followed by a Tukey multiple comparisons posttest

In the following experiments, we mainly investigated the ability of miR-29b to influence the VM formation of CRC cells. The blank, NC, and miR-29b inhibitor + si-ETV4 groups exhibited the ability of cells to form a circular structure after culture, even though the tube-shaped VM was not completely formed. In the miR-29b mimic and siRNA-ETV4 groups, cell proliferation, migration, and deformability were significantly attenuated, and less VM was formed. We also discovered a significant tubular VM formation in the miR-29b inhibitor group (Fig. 9a, b). When compared with the blank (11.67 ± 1.53) and NC (10.67 ± 1.53) groups, VM density was significantly lower in the miR-29b mimic (7.00 ± 1.00) and siRNA-ETV4 (6.33 ± 0.58) groups. Furthermore, the VM density in the miR-29b inhibitor group (16.00 ± 1.00) was significantly increased, whereas no significant changes were observed in the miR-29b inhibitor + si-ETV4 group (12.33 ± 1.15). Compared with the DMSO group (12.00 ± 1.00), the U0126 group showed reduced VM density (7.33 ± 0.58). These above-reported findings suggested that up-regulated miR-29b expression suppressed the VM formation of CRC cells.

### Up-regulated miR-29b impedes CRC cell migration and invasion through down-regulation of ETV4

**Fig. 10:**
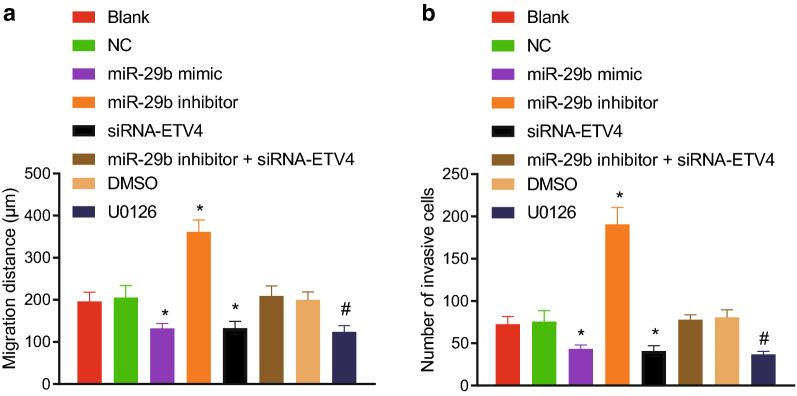
Up-regulated miR-29b impedes CRC cell migration and invasion. **a** The migration distance of CRC cells transfected with miR-29b mimic, miR-29b inhibitor and/or siRNA-ETV4. B, The number of invasive cells transfected with miR-29b mimic, miR-29b inhibitor and/or siRNA-ETV4. *p < 0.05 compared with the blank and NC groups. ^#^*p* < 0.05 compared with the DMSO group. Each experiment was repeated 3 times independently. Data among multiple groups were analyzed by one-way ANOVA, followed by a Tukey multiple comparisons post-test

To study the effect of miR-29b on cell migration of CRC, we conducted a scratch test. As depicted in Fig. 10a, there was no significant difference in the migration distance between the blank and NC groups. Compared with the blank and NC groups, the migration distance was notably shorter both in the miR-29b mimic and siRNA-ETV4 groups, respectively, however, it was significantly increased in the miR-29b inhibitor group. There was no significant difference in cell migration between the miR-29b inhibitor + siRNA-ETV4 group and the NC group. However, in relation to the DMSO group, the migration distance was lower in the U0126 group. Additionally, Transwell assay results exhibited no significant difference in cell invasion between the blank and NC groups (Fig. 10b). Compared with the blank and NC groups, cell invasion was markedly decreased in the miR-29b mimic and siRNA-ETV4 groups, but was greatly increased in the miR-29b inhibitor group, with no changes observed in the miR-29b inhibitor + siRNA-ETV4 group. In relation to the DMSO group, cell invasion was reduced in the U0126 group. These results strongly suggested that up-regulated miR-29b inhibited the CRC cell migration and invasion by targeting the ETV4.

### Up-regulated miR-29b expression arrests cells in the G0/G1 phase and promotes cell apoptosis through down-regulation of ETV4

**Fig. 11:**
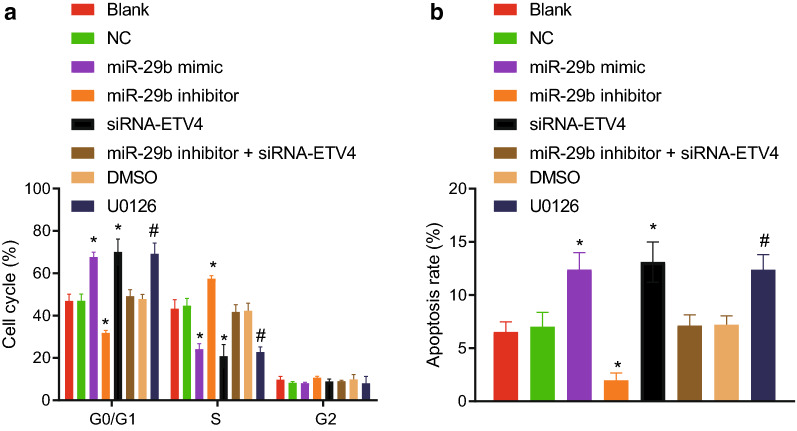
Up-regulated miR-29b arrests cells at the G0/G1 phase and promotes CRC cell apoptosis. **a** Cell cycle distribution assessed PI single staining. **b** Cell apoptosis detected by Annexin V-FITC and PI double staining. *p < 0.05 compared with the blank and NC groups. ^#^*p* < 0.05 compared with the DMSO group. Each experiment was repeated 3 times independently. Data among multiple groups were analyzed by one-way ANOVA, followed by a Tukey multiple comparisons posttest

Finally, we evaluated the effect of miR-29b on the CRC cell cycle and apoptosis using flow cytometry, with PI staining to evaluate the cell cycle distribution. As illustrated in Fig. 11a, there was no significant difference regarding cell proportions in different phases in the blank, NC, and miR-29b inhibitor + siRNA-ETV4 groups. However, we observed that increased number of cells was arrested at the G0/G1 phase but fewer cells were arrested at the S phase in the miR-29b mimic and siRNA-ETV4 groups. Moreover, there were fewer G0/G1 phase-arrested cells and more S G2 phase-arrested cells in the miR-29b inhibitor group in comparison with the blank and NC groups (all *p* < 0.05). Compared with the DMSO group, the U0126 group showed more G0/G1 phase-arrested cells and fewer S G2 phase-arrested cells.

Annexin V-FITC/PI double staining method was used to determine the apoptosis of CRC cells (Fig. 11b). However, there was no significant difference in apoptosis rate between the blank and NC groups. Compared with the blank and NC groups, miR-29b mimic and siRNA-ETV4 groups manifested increased apoptosis rate, while the miR-29b inhibitor group showed reduced apoptosis rate. There was no notable difference in apoptosis rate between the miR-29b inhibitor + siRNA-ETV4 groups and the NC group. Compared with the DMSO group, there was an increased apoptosis rate in the U0126 group.

Moreover, we found that the promoted cell cycle progression but inhibited cell apoptosis caused by inhibition of miR-29b was counteracted by the siRNA-mediated silencing of ETV4. These results collectively suggested that overexpression of miR-29b down-regulated expression of ETV4 to restrain cell cycle progression and to promote the CRC cell apoptosis.

### ETV4 mediated activation of ERK signaling pathway via regulating EGFR expression

**Fig. 12:**
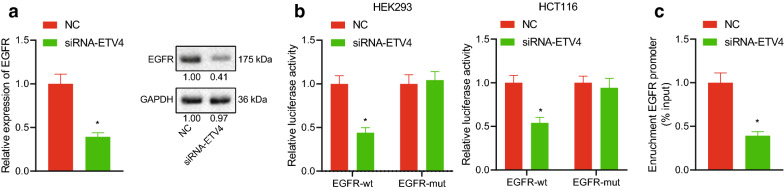
ETV4 promoted EGFR transcription to activate ERK signaling pathway. **a** Expression of EGFR mRNA and protein in cells detected by RT-qPCR and Western blot. **b** The binding of ETV4 to EGFR promoter in HEK293 and HCT116 cells detected by Luciferase assay. **c** The binding of ETV4 to EGFR promoter by CHIP. *p < 0.05 compared with the NC group, MUT, or IgG. Each experiment was repeated 3 times independently. Data between the two groups were compared by unpaired *t*-test

After ETV4 was silenced in HCT116 cells, expression of EGFR mRNA and protein was detected by RT-qPCR and Western blot. The results showed that, compared with the NC group, expression of EGFR mRNA and protein in the siRNA-ETV4 group was significantly decreased (Fig. 12a). Luciferase assay was used to detect luciferase activity in HEK293 and CRC HCT116 cells. The results (Fig. 12b) showed that luciferase activity of EGFR-WT decreased significantly after ETV4 interference. CHIP results showed that ETV4 protein could enrich the promoter of EGFR in HCT116 cells (Fig. 12c). In conclusion, the results showed that the transcription factor ETV4 could bind to the promoter region of EGFR to promote its transcription and positively regulate expression of EGFR. We speculated that ETV4 promoted EGFR transcription to activate ERK signaling pathway.

## Discussion

**Fig. 13:**
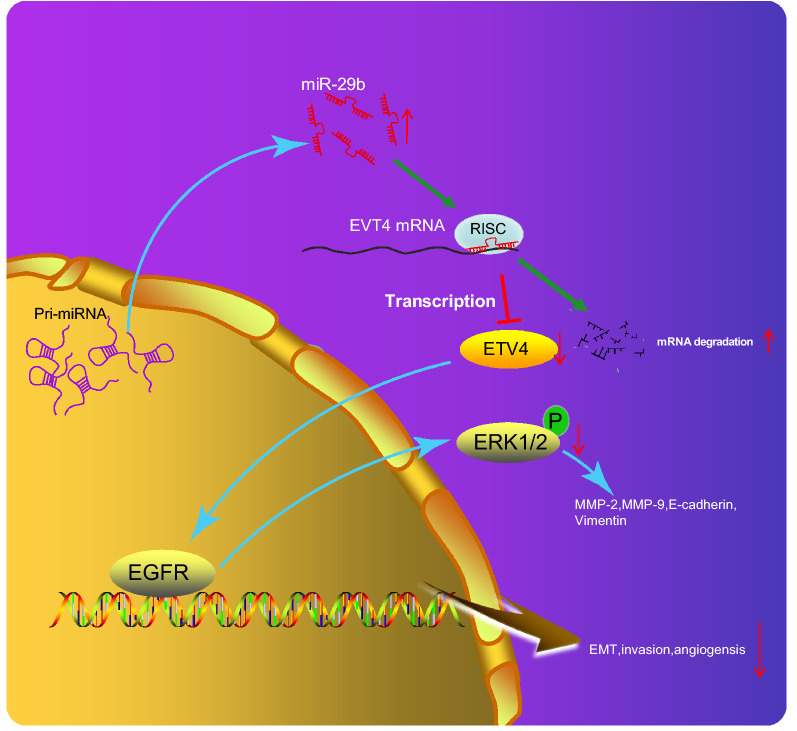
The graphical summary of the function and mechanism of miR-29b in CRC. miR-29b inhibits expression of ETV4 and EGFR transcription, consequently disrupted activation of the ERK signaling pathway, thus suppressing EMT and angiogenesis in CRC

Recent studies have highlighted that the differential expression of miRNAs could activate and regulate several signaling pathways, such as the activation of the ERK signaling pathway by miR-29b [37, 38]. Intriguingly, the results in the present study suggested that up-regulated miR-29b could potentially reduce expression of ETV4 and EGFR transcription and block the activation of the ERK signaling pathway, thereby preventing EMT and angiogenesis of CRC (Fig. 13).

First, our RT-qPCR results showed poorer expression of miR-29b in CRC tissues than in adjacent normal tissues. Similarly, the serum levels of miR-29b were lower in CRC patients than in control subjects. We also found that miR-29b expression was inversely correlated with the degree of TNM staging, as previously reported [39]. Importantly, our investigation further determined that low expression of miR-29b was associated with LNM, poor differentiation grade, and advanced TNM stage in patients with CRC. This is consistent with the previous observations that miR-29b could inhibit tumor growth and metastasis in CRC by suppressing EMT [7]. Our study further verifies that miR-29b could suppress the angiogenesis and EMT in CRC. Moreover, our study highlighted miR-29b as a tumor-suppressive miRNA via its ability to suppress cell proliferation, migration, and invasion, while promoting cell apoptosis in CRC. Additionally, these findings were further verified at the molecular level by up-regulated E-cadherin and TSP-1 levels, and reduced levels of MMP-2, MMP-9, Vimentin, and VEGF. Nevertheless, MMP-2 and MMP-9 are MMPs, which are involved in the VEGF-mediated angiogenesis and MMP-mediated endoglin (a neovascularization marker), and which have elsewhere been reported to promote the angiogenic features of endothelial cells in CRC [40]. Indeed, stromal Vimentin expression has the potential to be a promising indicator for survival prediction and adjuvant chemotherapy response in high-risk stage II CRC patients [41]. A previous study has reported that miR-29b suppresses angiogenesis by targeting of MMP-2 and VEGFA [42], although restoration of miR-29b could inhibit the metastasis and reverse the EMT in bladder cancer [43]. However, ectopic expression of miR-29b has been indicated to induce this reversal of EMT and aid in tube formation in vitro by CRC cell lines, i.e., SW480 [44]. Moreover, miR-29b has been illustrated to impede CRC cell proliferation but facilitate cell apoptosis [8]. Hence, these above-described findings are in some respects consistent with our data. Thus, we are confident in proposing that miR-29b could inhibit CRC development and progression by inhibiting cell growth, metastasis, and angiogenesis, as well as promoting cell apoptosis.

ETV4 protein is documented to be significantly up-regulated in CRC patients as well as in CRC cell lines [12], which we recapitulated in the present immunohistochemistry, RT-qPCR, and Western blot analysis. Generally speaking, miRNAs can regulate gene expression post-transcriptionally by interacting with the 3’UTR of specific target mRNAs [45]. Emerging evidence demonstrates that miRNAs play an important role in regulating cancer cell growth, invasion, and metastasis by inhibiting expression of their targets [46]. Our present study ascertained that ETV4 was a target gene of miR-29b, such that elevated miR-29b could down-regulate ETV4 expression, which might be the potential mechanism responsible for the anti-oncogenic role of miR-29b. ETS genes may also serve as potential markers of cancer invasiveness and metastasis. ETV4, which is a member of ETS family, is elevated in CRC, and to an especially great extent in the event of lymph node involvement [47]. Other research implicates ETV4 expression in lymphatic and venous invasion, LNM and distant metastasis, advanced pathological TNM stage and even tumor relapse [48], which was in consistent with our findings that high expression of ETV4 was associated with LNM, poor differentiation grade and advanced TNM stage in patients with CRC. ETS1, another ETS member, affects disease in patients with non-small-cell lung cancer *via* up-regulation of miR-29b expression in the immune-evasion subtype [9]. Additionally, ETV4 can act as an oncogenic protein to promote the development and progression of CRC [49], as seen in other research revealing that ETV4 is associated with overexpressed MMPs in CRC [50]. Previous work shows that siRNA-mediated knockdown of ETV4 results in reduced colon cancer cell proliferation and invasion [51]. Similarly, in our study, CRC cells transfected with siRNA-ETV4 exhibited diminished cell viability, migration and invasion abilities, as well as inhibited EMT and angiogenesis. Our rescue experiments further demonstrated that the promotion of cancer progression induced by miR-29b inhibitor was neutralized by siRNA-ETV4, suggesting that inhibition of ETV4 conferred the suppressive role of miR-29b in CRC progression.

Other research documents that siRNA-mediated knockdown of ETV4 results in reduced cancer cell proliferation and invasion [51]. Consistently, our study indicated that CRC cells transfected with siRNA-ETV4 exhibited diminished cell viability, migration, and invasion abilities as well as inhibited the EMT and angiogenesis. Our present rescue experiments further confirmed that siRNA-ETV4 neutralized the promotion of cancer progression induced by miR-29b inhibitor, suggesting that inhibition of ETV4 conferred a suppressive role of miR-29b in CRC progression.

Furthermore, our results exhibited that miR-29b could suppress the activation of the ERK signaling pathway *via* ETV4 and EGFR down-regulation in CRC, consistent with earlier findings showing that the ERK signaling pathway can repress tumor angiogenesis and tumor growth [52] and attributing inactivation of the ERK signaling pathway to ETV4 gene silencing [35]. Moreover, based on other previous literature reports [53, 54], EGFR is located upstream of the ERK signaling pathway, which activates ERK signaling pathway in tumor to promote tumor development. Yuan et al.. have reported that miR-29b suppressed CRC cell growth and invasion by inhibiting insulin-like growth factor 1 (IGF1), an activator of the phosphatidylinositol 3-kinase/protein kinase B (PI3K/Akt) signaling pathway [55]. However, the authors of that study proposed a positive feedback loop between IRF1 and miR-29b, which we did not test in the present study. Taken together, our results showed that increased expression of miR-29b resulted in down-regulation of ETV4, which led to the suppression of EMT and angiogenesis in CRC cells *via* blocking the ERK signaling pathway.

## Conclusions

In summary, we demonstrated that overexpression of miR-29b could suppress the EMT and angiogenesis in CRC via disrupting the ETV4-dependent activation of the ERK signaling pathway (Fig. 13). These findings may provide further insights into the clinical application of miRNA-targeted therapies for CRC. Although the complex factors and signaling pathways involving EMT and angiogenesis remain poorly understood, however, we give some new insight into the specific molecular mechanisms mediating the anti-tumor effect of miR-29b-targeted inhibition of ETV4 in CRC.

**Abbreviations** .

CRC, colorectal cancer; miR-29, microRNA-29; miRs or miRNAs, microRNAs; ETS1, E26 transformation-specific-1; ETV4, ETS variant 4; GEO, Gene Expression Omnibus; ERK, signal-regulated kinase; DEGs, differentially expressed genes; PPI, protein-protein interaction; AJCC, American Joint Committee on Cancer; TNM, tumor node metastasis; EMT, epithelial-mesenchymal transition; HE, hematoxylin-eosin; PBS, phosphate buffered saline; BSA, bovine serum albumin; IgG, immunoglobulin G; DAB, diaminobenzidine; TRG, tumor regression grade; SP, streptavidin-perosidase; MVD, micro-vessel density; DMEM, Dulbecco’s modified Eagle’s medium; PAS, Periodic acid-Shiff’s; Opti-MEM, Opti-minimum essential medium; FBS, fetal bovine serum; NC, negative control; siRNA, small interfering RNA; 3’-UTR, 3’-untranslated region; WT, wild-type; MUT, mutant; RT-qPCR, reverse transcription quantitative polymerase chain reaction; cDNA, complementary DNA; MMP, matrix metalloproteinase; VEGF, vascular endothelial growth factor; GAPDH, glyceraldehyde-3-phosphate dehydrogenase; RIPA, radioimmunoprecipitation assay; FFPE, formalin-fixed paraffin-embedded; BCA, bicinchoninic acid; PAGE, polyacrylamide gel electrophoresis; NC, nitrocellulose; TBST, Tris-buffered saline Tween-20; ECL, enhanced chemiluminescence; MTT, 3-(4,5-dimethylthiazol-2-yl)-2, 5-diphenyltetrazolium bromide; DMSO, dimethyl sulfoxide; OD, optical density; VM, vasculogenic mimicry; PI, propidium iodide; FITC, fluorescein isothiocyanate; ANOVA, analysis of variance; IGF1, insulin-like growth factor 1; PI3K/Akt, phosphatidylinositol 3-kinase/protein kinase B.

## Data Availability

The datasets used and analyzed during the current study are available from the corresponding author on reasonable request.

## Abbrevations

CRC: Colorectal cancer
miR-29: microRNA-29
miRs or miRNAs: microRNAs
ETS1: E26 transformation-specific-1
ETV4: ETS variant 4
GEO: Gene Expression Omnibus
ERK: Signal-regulated kinase
DEGs: Differentially expressed genes
PPI: Protein–protein interaction
AJCC: American Joint Committee on Cancer
TNM: Tumor node metastasis
EMT: Epithelial-mesenchymal transition
HE: Hematoxylin-eosin
PBS: Phosphate buffered saline
BSA: Bovine serum albumin
IgG: Immunoglobulin G
DAB: Diaminobenzidine
TRG: Tumor regression grade
SP: Streptavidin-perosidase
MVD: Micro-vessel density
DMEM: Dulbecco’s modified Eagle’s medium
PAS: Periodic acid-Shiff’s
Opti-MEM: OPTI-minimum essential medium
FBS: Fetal bovine serum
NC: Negative control
siRNA: Small interfering RNA
3′-UTR: 3′-Untranslated region
WT: Wild-type
MUT: Mutant
RT-qPCR: Reverse transcription quantitative polymerase chain reaction
cDNA: Complementary DNA
MMP: Matrix metalloproteinase
VEGF: Vascular endothelial growth factor
GAPDH: Glyceraldehyde-3-phosphate dehydrogenase
RIPA: Radioimmunoprecipitation assay
FFPE: Formalin-fixed paraffin-embedded
BCA: Bicinchoninic acid
PAGE: Polyacrylamide gel electrophoresis
NC: Nitrocellulose
TBST: Tris-buffered saline Tween-20
ECL: Enhanced chemiluminescence
MTT: 3-(4,5-Dimethylthiazol-2-yl)-2, 5-diphenyltetrazolium bromide
DMSO: Dimethyl sulfoxide
OD: Optical density
VM: Vasculogenic mimicry
PI: Propidium iodide
FITC: Fluorescein isothiocyanate
ANOVA: Analysis of variance
IGF1: Insulin-like growth factor 1
PI3K/Akt: Phosphatidylinositol 3-kinase/protein kinase B

## References

[1] Bray F, Ferlay J, Soerjomataram I, Siegel RL, Torre LA, Jemal A (2018). Global cancer statistics 2018: GLOBOCAN estimates of incidence and mortality worldwide for 36 cancers in 185 countries. CA Cancer J Clin.

[2] Brenner H, Kloor M, Pox CP (2014). Colorectal cancer. Lancet.

[3] Abernathy K, Abernathy Z, Brown K, Burgess C, Hoehne R (2017). Global dynamics of a colorectal cancer treatment model with cancer stem cells. Heliyon.

[4] Grosso G, Biondi A, Galvano F, Mistretta A, Marventano S, Buscemi S, Drago F, Basile F (2014). Factors associated with colorectal cancer in the context of the Mediterranean diet: a case-control study. Nutr Cancer.

[5] Cervigne NK, Reis PP, Machado J, Sadikovic B, Bradley G, Galloni NN, Pintilie M, Jurisica I, Perez-Ordonez B, Gilbert R (2009). Identification of a microRNA signature associated with progression of leukoplakia to oral carcinoma. Hum Mol Genet.

[6] Monteleone NJ, Moore AE, Iacona JR, Lutz CS, Dixon DA (2019). miR-21-mediated regulation of 15-hydroxyprostaglandin dehydrogenase in colon cancer. Sci Rep.

[7] Wang B, Li W, Liu H, Yang L, Liao Q, Cui S, Wang H, Zhao L (2014). miR-29b suppresses tumor growth and metastasis in colorectal cancer via downregulating Tiam1 expression and inhibiting epithelial-mesenchymal transition. Cell Death Dis.

[8] Inoue A, Yamamoto H, Uemura M, Nishimura J, Hata T, Takemasa I, Ikenaga M, Ikeda M, Murata K, Mizushima T (2015). MicroRNA-29b is a novel prognostic marker in colorectal cancer. Ann Surg Oncol.

[9] Taylor MA, Wappett M, Delpuech O, Brown H, Chresta CM (2016). Enhanced MAPK signaling drives ETS1-mediated induction of miR-29b leading to downregulation of TET1 and changes in epigenetic modifications in a subset of lung SCC. Oncogene.

[10] Zhang XM, Guo L, Huang X, Li QM, Chi MH (2016). 4-Hydroxynonenal regulates TNF-alpha gene transcription indirectly via ETS1 and microRNA-29b in human adipocytes induced from adipose tissue-derived stromal cells. Anat Rec (Hoboken).

[11] Dumortier M, Ladam F, Damour I, Vacher S, Bieche I, Marchand N, de Launoit Y, Tulasne D, Chotteau-Lelievre A (2018). ETV4 transcription factor and MMP13 metalloprotease are interplaying actors of breast tumorigenesis. Breast Cancer Res.

[12] Xiao J, Yang S, Shen P, Wang Y, Sun H, Ji F, Zhou D (2017). Phosphorylation of ETV4 at Ser73 by ERK kinase could block ETV4 ubiquitination degradation in colorectal cancer. Biochem Biophys Res Commun.

[13] Dissanayake K, Toth R, Blakey J, Olsson O, Campbell DG, Prescott AR, MacKintosh C (2011). ERK/p90(RSK)/14-3-3 signalling has an impact on expression of PEA3 Ets transcription factors via the transcriptional repressor capicua. Biochem J.

[14] Liu J, Xiao X, Shen Y, Chen L, Xu C, Zhao H, Wu Y, Zhang Q, Zhong J, Tang Z (2017). MicroRNA-32 promotes calcification in vascular smooth muscle cells: Implications as a novel marker for coronary artery calcification. PLoS ONE.

[15] Gautier L, Cope L, Bolstad BM, Irizarry RA (2004). affy–analysis of Affymetrix GeneChip data at the probe level. Bioinformatics.

[16] Smyth GK (2004). Linear models and empirical bayes methods for assessing differential expression in microarray experiments. Stat Appl Genet Mol Biol.

[17] Bardou P, Mariette J, Escudie F, Djemiel C, Klopp C (2014). jvenn: an interactive Venn diagram viewer. BMC Bioinformatics.

[18] Shannon P, Markiel A, Ozier O, Baliga NS, Wang JT, Ramage D, Amin N, Schwikowski B, Ideker T (2003). Cytoscape: a software environment for integrated models of biomolecular interaction networks. Genome Res.

[19] Amin MB, Greene FL, Edge SB, Compton CC, Gershenwald JE, Brookland RK, Meyer L, Gress DM, Byrd DR, Winchester DP (2017). The Eighth Edition AJCC Cancer Staging Manual: Continuing to build a bridge from a population-based to a more “personalized” approach to cancer staging. CA Cancer J Clin.

[20] Erarslan E, Turkay C, Koktener A, Koca C, Uz B, Bavbek N (2009). Association of visceral fat accumulation and adiponectin levels with colorectal neoplasia. Dig Dis Sci.

[21] Singsuksawat E, Thuwajit C, Charngkaew K, Thuwajit P (2018). Increased ETV4 expression correlates with estrogen-enhanced proliferation and invasiveness of cholangiocarcinoma cells. Cancer Cell Int.

[22] Chen WC, Lu YC, Kuo SJ, Lin CY, Tsai CH, Liu SC, Chen YL, Wang SW, Tang CH (2020). Resistin enhances IL-1beta and TNF-alpha expression in human osteoarthritis synovial fibroblasts by inhibiting miR-149 expression via the MEK and ERK pathways. FASEB J..

[23] Zhao J, Fang Z, Zha Z, Sun Q, Wang H, Sun M, Qiao B (2019). Quercetin inhibits cell viability, migration and invasion by regulating miR-16/HOXA10 axis in oral cancer. Eur J Pharmacol.

[24] Gu TT, Chen TY, Yang YZ, Zhao XJ, Sun Y, Li TS, Zhang DM, Kong LD (2019). Pterostilbene alleviates fructose-induced renal fibrosis by suppressing TGF-beta1/TGF-beta type I receptor/Smads signaling in proximal tubular epithelial cells. Eur J Pharmacol.

[25] Li H, Meng X, Zhang D, Xu X, Li S, Li Y (2019). Ginkgolic acid suppresses the invasion of HepG2 cells via downregulation of HGF/cMet signaling. Oncol Rep.

[26] Wang XH, Wu HY, Gao J, Wang XH, Gao TH, Zhang SF (2019). FGF represses metastasis of neuroblastoma regulated by MYCN and TGF-beta1 induced LMO1 via control of let-7 expression. Brain Res.

[27] Zhao F, Yang X, Xu G, Bi J, Lv R, Huo R (2019). Propranolol suppresses HUVEC viability, migration, VEGF expression, and promotes apoptosis by downregulation of miR-4295. J Cell Biochem.

[28] Qu S, Yang L, Liu Z (2020). MicroRNA-194 reduces inflammatory response and human dermal microvascular endothelial cells permeability through suppression of TGF-beta/SMAD pathway by inhibiting THBS1 in chronic idiopathic urticaria. J Cell Biochem.

[29] Yuan YH, Wang HY, Lai Y, Zhong W, Liang WL, Yan FD, Yu Z, Chen JK, Lin Y (2019). Epigenetic inactivation of HOXD10 is associated with human colon cancer via inhibiting the RHOC/AKT/MAPK signaling pathway. Cell Commun Signal.

[30] Wu N, Zhao X, Liu M, Liu H, Yao W, Zhang Y, Cao S, Lin X (2011). Role of microRNA-26b in glioma development and its mediated regulation on EphA2. PLoS ONE.

[31] Sun Q, Zou X, Zhang T, Shen J, Yin Y, Xiang J (2014). The role of miR-200a in vasculogenic mimicry and its clinical significance in ovarian cancer. Gynecol Oncol.

[32] Wang J, Hollingshead J, El-Masry N, Horncastle D, Talbot I, Tomlinson I, Alison MR, El-Bahrawy M (2012). Expression of EGFR, HER2, phosphorylated ERK and phosphorylated MEK in colonic neoplasms of familial adenomatous polyposis patients. J Gastrointest Cancer.

[33] Xu T, Zhou M, Peng L, Kong S, Miao R, Shi Y, Sheng H, Li L (2014). Upregulation of CD147 promotes cell invasion, epithelial-to-mesenchymal transition and activates MAPK/ERK signaling pathway in colorectal cancer. International Journal of Clinical Experimental Pathology.

[34] Ye Q, Cai W, Zheng Y, Evers BM, She QB (2014). ERK and AKT signaling cooperate to translationally regulate survivin expression for metastatic progression of colorectal cancer. Oncogene.

[35] Fontanet P, Irala D, Alsina FC, Paratcha G, Ledda F (2013). Pea3 transcription factor family members Etv4 and Etv5 mediate retrograde signaling and axonal growth of DRG sensory neurons in response to NGF. J Neurosci.

[36] Keld R, Guo B, Downey P, Cummins R, Gulmann C, Ang YS, Sharrocks AD (2011). PEA3/ETV4-related transcription factors coupled with active ERK signalling are associated with poor prognosis in gastric adenocarcinoma. Br J Cancer.

[37] Chen HX, Xu XX, Tan BZ, Zhang Z, Zhou XD (2017). MicroRNA-29b Inhibits Angiogenesis by Targeting VEGFA through the MAPK/ERK and PI3K/Akt Signaling Pathways in Endometrial Carcinoma. Cell Physiol Biochem.

[38] Park EC, Kim G, Jung J, Wang K, Lee S, Jeon SS, Lee ZW, Kim SI, Kim S, Oh YT (2013). Differential expression of MicroRNAs in patients with glioblastoma after concomitant chemoradiotherapy. OMICS.

[39] Basati G, Razavi AE, Pakzad I, Malayeri FA (2016). Circulating levels of the miRNAs, miR-194, and miR-29b, as clinically useful biomarkers for colorectal cancer. Tumor Biology.

[40] Araujo RF, Lira GA, Vilaca JA, Guedes HG, Leitao MC, Lucena HF, Ramos CC (2015). Prognostic and diagnostic implications of MMP-2, MMP-9, and VEGF-alpha expressions in colorectal cancer. Pathol Res Pract.

[41] Liu LG, Yan XB, Xie RT, Jin ZM, Yang Y (2017). Stromal Expression of Vimentin Predicts the Clinical Outcome of Stage II Colorectal Cancer for High-Risk Patients. Med Sci Monit.

[42] Li P, Guo W, Du L, Zhao J, Wang Y, Liu L, Hu Y, Hou Y (2013). microRNA-29b contributes to pre-eclampsia through its effects on apoptosis, invasion and angiogenesis of trophoblast cells. Clin Sci.

[43] Lv M, Zhong Z, Huang M, Tian Q, Jiang R, Chen J (2017). lncRNA H19 regulates epithelial-mesenchymal transition and metastasis of bladder cancer by miR-29b-3p as competing endogenous RNA. Biochim Biophys Acta Mol Cell Res.

[44] Subramanian M, Rao SR, Thacker P, Chatterjee S, Karunagaran D (2014). MiR-29b downregulates canonical Wnt signaling by suppressing coactivators of beta-catenin in human colorectal cancer cells. J Cell Biochem.

[45] Ivey KN, Srivastava D (2015). microRNAs as Developmental Regulators. Cold Spring Harb Perspect Biol.

[46] Chan SH, Wang LH (2015). Regulation of cancer metastasis by microRNAs. J Biomed Sci.

[47] Deves C, Renck D, Garicochea B, da Silva VD, Giulianni Lopes T, Fillman H, Fillman L, Lunardini S, Basso LA, Santos DS (2011). Analysis of select members of the E26 (ETS) transcription factors family in colorectal cancer. Virchows Arch.

[48] Horiuchi S, Yamamoto H, Min Y, Adachi Y, Itoh F, Imai K (2003). Association of ets-related transcriptional factor E1AF expression with tumour progression and overexpression of MMP-1 and matrilysin in human colorectal cancer. J Pathol.

[49] Yuan ZY, Dai T, Wang SS, Peng RJ, Li XH, Qin T, Song LB, Wang X (2014). Overexpression of ETV4 protein in triple-negative breast cancer is associated with a higher risk of distant metastasis. OncoTargets Therapy.

[50] Sha JJ, Dong YH, Liu DM, Bo JJ, Huang YR, Li Z, Ping P (2013). Regulation network analysis of testicular seminoma at various stages of progression. Genet Mol Res.

[51] Moss AC, Lawlor G, Murray D, Tighe D, Madden SF, Mulligan AM, Keane CO, Brady HR, Doran PP, MacMathuna P (2006). ETV4 and Myeov knockdown impairs colon cancer cell line proliferation and invasion. Biochem Biophys Res Commun.

[52] Iskender B, Izgi K, Canatan H (2016). Novel anti-cancer agent myrtucommulone-A and thymoquinone abrogate epithelial-mesenchymal transition in cancer cells mainly through the inhibition of PI3K/AKT signalling axis. Mol Cell Biochem.

[53] Fan Y, Xue W, Schachner M, Zhao W (2018). Honokiol eliminates glioma/glioblastoma stem cell-like cells via JAK-STAT3 Signaling and inhibits tumor progression by targeting epidermal growth factor receptor. Cancers.

[54] Moreno E, Valon L, Levillayer F, Levayer R (2019). Competition for space induces cell elimination through compaction-driven ERK downregulation. Curr Biol.

[55] Yuan L, Zhou C, Lu Y, Hong M, Zhang Z, Zhang Z, Chang Y, Zhang C, Li X (2015). IFN-gamma-mediated IRF1/miR-29b feedback loop suppresses colorectal cancer cell growth and metastasis by repressing IGF1. Cancer Lett.

